# Lilikoi: an R package for personalized pathway-based classification modeling using metabolomics data

**DOI:** 10.1093/gigascience/giy136

**Published:** 2018-12-10

**Authors:** Fadhl M AlAkwaa, Breck Yunits, Sijia Huang, Hassam Alhajaji, Lana X Garmire

**Affiliations:** 1 Department of Computational Medicine and Bioinformatics, Building 520, 1600 Huron Parkway, Ann Arbor, MI 48109, USA; 2 University of Hawaii Cancer Center, Department of Epidemiology, 701 Ilalo Street, Honolulu, HI USA 96813; 3 Molecular Biology and Bioengineering Graduate Program, University of Hawaii at Monoa, Honolulu, HI, USA 96822

**Keywords:** metabolomics, pathway, classification, feature selection, machine learning, mapping

## Abstract

Lilikoi (the Hawaiian word for passion fruit) is a new and comprehensive R package for personalized pathway-based classification modeling using metabolomics data. Four basic modules are presented as the backbone of the package: feature mapping module, which standardizes the metabolite names provided by users and maps them to pathways; dimension transformation module, which transforms the metabolomic profiles to personalized pathway-based profiles using pathway deregulation scores; feature selection module, which helps to select the significant pathway features related to the disease phenotypes; and classification and prediction module, which offers various machine learning classification algorithms. The package is freely available under the GPLv3 license through the github repository at: https://github.com/lanagarmire/lilikoi and CRAN: https://cran.r-project.org/web/packages/lilikoi/index.html.

## Introduction

Metabolomics has been increasingly employed as a systematic approach to investigate the relationship between cellular signals and phenotypes [1]. Non-targeted metabolomics with global measurements helps to discover novel metabolite biomarkers for diseases and conditions [2]. However, due to factors such as non-standardized protocols and highly heterogeneous study populations, it is difficult to find robust biomarkers that can be translated into clinical applications [3, 4].

Currently, there are multitudes of secondary metabolomics analysis tools, primarily in the form of web tools. Very few comprehensive packages exist in R/Bioconductor, the dominant bioinformatics scripting language, in order to support metabolomics data analysis. Various modules of metabolomics pipelines have been implemented in other programming languages, including preprocessing [5], compound mapping [6], pathway networks [7], visualization [8], deep learning [9], and statistical enrichment analysis [10].

In the pathway analysis area, various approaches have been proposed to analyze metabolomics data, such as MetPA [11], IMPaLA [12], and MPEA [13]. The common feature of these methods is that they use metabolites as biological entities to summarize to pathway-level statistics at the group level (separated by states) and then perform enrichment analysis (such as over-representation analysis and gene set enrichment analysis [GSEA]) in order to calculate the over-representation of pathways in one group vs the other group. As a result, over-representation of pathways in one group vs the other group is estimated. Specifically, MetPA is a web tool that combines pathway enrichment analysis with pathway topological characteristics to help identify the most relevant metabolic pathways. IMPaLA is a web tool that performs joint pathway analysis of transcriptomics or proteomics and metabolomics data through over-representation or enrichment analysis. MPEA is another pathway analysis tool based on GSEA principles, designed specifically to handle many-to-many relationships that may occur between the query compounds and metabolite annotations. However, none of these pathway-based methods transform the metabolite-sample matrix into pathway-sample matrix in order to entail pathway representation at the individual sample level (or personalized level). Moreover, these pathway-based methods are generally used as system biology-level interpretation of metabolomics, and they are incapable of constructing pathway features, upon which classification algorithms are built for the purpose of biomarker modeling.

To fill the void above, we introduce a new R package called Lilikoi (the Hawaiian name for passion fruit), which specializes in personalized pathway measurement and classification prediction models. We present this tool in four modules: feature-pathway mapper, which standardizes metabolite ID and maps them to pathways; dimension transformation, which derives personalized pathway deregulation scores from metabolite profiles; feature selection, which provides the user with a range of feature selection algorithms to select significant features related to phenotypes; classification and prediction, which lists a series of classification algorithms to derive machine learning models and give predictions on testing datasets.

## Methods

### Overall design of Lilikoi

**Figure 1:**
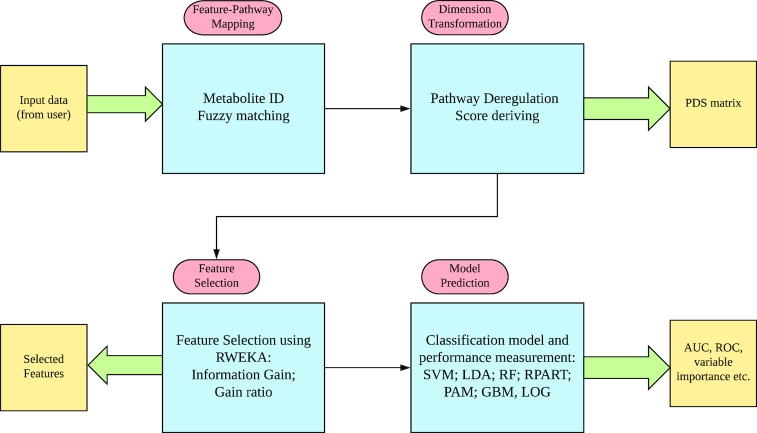
The workflow of Lilikoi package. Lilikoi is composed of four modules: feature mapper; dimension transformer; feature selector; and classification predictor.

The Lilikoi package can be divided into four functional modules (Fig. 1): feature mapper, dimension transformer, feature selector, and classification predictor. In the first module, Lilikoi takes metabolite profile data from the user as the input feature and standardizes the metabolite names to various IDs in databases including Kyoto Encyclopedia of Genes and Genomes (KEGG), PubChem, Human Metabolome Database (HMDB), and Metabolite and Tandom Mass Spectrometry (METLIN). After the mapping step, the second module transforms metabolite profiles to a comprehensive pathway deregulation score (PDS) matrix based on the *Pathifier* algorithm [14]. The third module employs various feature selection algorithms to select key pathway *features* in the training set that are significantly related to phenotypes. The final classification module builds a classification model on the training set based on various algorithms, including random forest (RF), support vector machine (SVM), linear discriminate analysis (LDA), logistic regression (LOG), prediction analysis for microarray (PAM), generalized boosted model (GBM), and recursive partitioning and regression analysis (RPART). It then performs prediction and quantitative evaluations on testing sets using various metrics. The details of each module are discussed in the following sections.

### Feature mapper

Reporting proper compounds with standard accession numbers is of paramount importance, and ownstream metabolomics data analysis is only possible with unique metabolite IDs. The non-standardized synonyms create many issues for data analysis and thus must be dealt with. A few tools have been developed to address this issue. The Chemical Translation Service is a web-based tool for metabolite ID conversion [15]. BridgeDb is another R package that supports gene, protein, and metabolite identifier mapping [16]. We implement feature mapper in Lilikoi, which embeds comprehensive databases including more than 18,000 metabolites and 100,000 synonyms. Lilikoi provides a default database and also allows updated database if the user prefers. The function lilikoi.updateDB() method allows users to call to pull the latest curated databases from Lilikoi's github repository.

**Figure 2:**
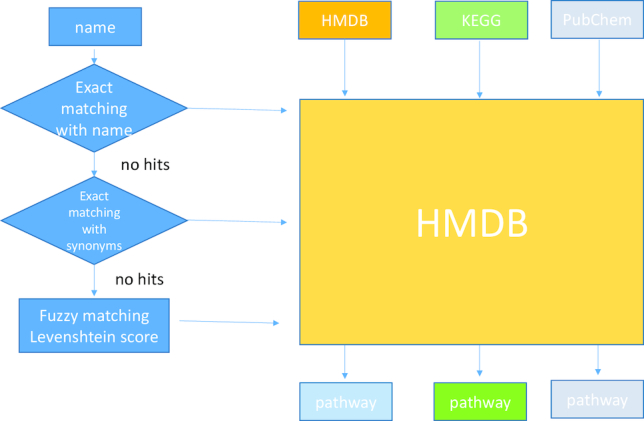
The workflow of module 1: feature mapper. The user can input any metabolite IDs such as chemical name, KEGG, PubChem, and HMDB IDs. The fuzzy matching algorithm is implemented to map the non-matched names to the 100k synonyms database.

The feature mapping process consists of three steps (Fig. 2). In step 1, the input metabolite names are mapped to HMDB IDs using exact matching. We include various databases such as HMDB, KEGG, PubChem, and MetaboAnalyst compound databases to standardize the metabolite names. In step 2, Lilikoi employs the synonym database to standardize the rest of the unmapped metabolites to HMDB IDs. The remaining unmapped metabolites go through the third fuzzy matching step. We calculate the Levenshtein edit distance as a measurement of string similarity and map the metabolite to the closest related standardized metabolites [17]. Such a process allows for maximal mapping of input metabolites to standardized HMDB IDs.

### Dimension transformation

Lilikoi applies the *Pathifier* algorithm to perform the metabolites-pathway dimension transformation [14]. This algorithm summarizes per-sample information from the metabolite level to the pathway level [14]. For each pathway, all samples are mapped to a high-dimensional principal component space (as data points), and a principal curve is constructed among them (the data cloud). A PDS score is then derived to measure the distance from the origin of the principle curve to the specific point on the principle curve, projected by the data point that represents a sample. The larger the PDS score, the farther a sample deviates from the normal level in that specific pathway. As the result of the dimension transformation step, a new pathway-level metabolomics profile matrix is constructed. The user can then use this matrix for downstream analysis. More details of applications of *Pathifier* on biomarker studies (prognosis or diagnosis) can be found in our earlier publications [4, 18].

The PDS score *D_P_(i)* was calculated for each pathway P and each sample *i*, based on the intensities of the metabolites in pathway P. This score estimates the extent to which the pathway P in sample *i* deviates from the control. Briefly, in the high-dimensional space *d_P_* made of metabolite vectors (where each metabolite belongs to pathway P), all samples form a data cloud, where sample *i* is a data point x_i_. The principle curve *S_P′_* in this space *d_P_* is then calculated using Hastie and Stuetzle's algorithm [19]. For each sample, the data point x_i_ is projected onto the principle curve *S_P′_*. The deregulation score *D_P_(i)* of sample *i* is then defined as the distance from the start of the principle curve to the projected point on this curve.

### Feature selection

Lilikoi allows the user to provide training and testing datasets, as well phenotype information for the samples. For the training set, Lilikoi provides two major feature selection algorithms: information gain (mutual information) and gain ratio, which select the most significant pathway-level features related to the phenotype. The *RWeka* package is required for the feature selection module [20]. Information gain statistic is provided to evaluate the added information from each feature to help discriminate the phenotype. Gain ratio statistic is an alternative metric that solves the problem of overfitting, when there are a large number of distinct variables. We recommend that the user uses the gain ratio instead of the information gain when the input dataset has categorical variables in addition to the metabolomics data. To assess overfitting, one can examine the difference in accuracy between the training and testing data. A much lower accuracy in testing data indicates overfitting.

### Classification and prediction

Seven widely used machine learning algorithms, including LDA, SVM, RF, RPART, PAM, LOG, and GBM, are supported by Lilikoi to build classification models. These methods have been widely used in the metabolomics community and reported in various research articles [9, 21–23]. Lilikoi uses R package *caret* for automatic parameter tuning of all the algorithms [24]. An n-fold (default n = 10; flexible depending on different sample sizes) cross-validation is applied on the training dataset to avoid overfitting. Metrics to measure prediction accuracy, including area under the curve (AUC), F1-statistic, balanced accuracy, sensitivity (SEN), and specificity (SPEC), are reported to the user as bar plots, similar to others [25]. Receiver operating characteristic (ROC) curves can also be reported as a separate figure.

To rank the importance of pathway features in the classification model, we used the variable importance function implemented in the *Caret* PR package. This function ranks features based on their contribution to the model performance.

### Combined model addressing confounding

The user can add any clinical factors such as age, sex, and ethnicity to the model. All of these factors are normalized between 0 and 1 by scaling between minimum and maximum values so that they are compatible with the PDS score.

### Example datasets

For demonstration, we present two metabolomics datasets. One set is from the City of Hope Hospital that was published earlier [4]. This dataset is composed of 207 samples from plasma (126 breast cancer cases and 81 controls). The details of the data are summarized in our previous work [4]. This dataset was downloaded from Metabolomics workbench [26] project ID PR000284. The second dataset consists of 271 breast cancer tissue samples (204 estrogen receptor [ER]+ and 67 ER−) collected from a biobank at the Pathology Department of Charité Hospital, Germany, as reported earlier [27]. The metabolomics profiles of these patients can be downloaded from the supplementary material of the study [28].

## Results

For illustration purposes, we applied Lilikoi to two metabolomics datasets of breast cancers. The first dataset is the plasma samples of breast cancer vs normal controls, which also have clinical information such as age, sex, and ethnicity [4]. The second dataset is the tissue samples of ER+ vs ER− breast cancer patients [27].

### Standardization and mapping of metabolomics IDs

A good practice of a metabolomics report is to have standardized identifiers. However, in reality, currently different metabolomics research laboratories/preprocessing tools generate metabolomics profiles using different naming standards, and this causes big problems for downstream bioinformatics analysis. To cope with it, Lilikoi first transforms the metabolite names to standard IDs. It allows the user to input any kind of metabolite IDs, their synonyms, KEGG IDs, HMDB IDs, or PubChem IDs. Moreover, Lilikoi embeds comprehensive databases, including more than 18,000 metabolites and 100,000 synonyms, in accordance with other types of input IDs. Another major user-friendly characteristic of Lilikoi is the implementation of a fuzzy matching algorithm, which allows better mapping of uncertain metabolites by calculating the string similarity score of the input metabolite name with those in the databases. These features of Lilikoi greatly improve its usability. In the first plasma sample set, 182 of 227 metabolites are mapped to standard HMDB IDs; in the second breast cancer tissue sample set, 120 of 162 metabolites are mapped to standard HMDB IDs.

### Metabolite to pathway level transformation

**Figure 3:**
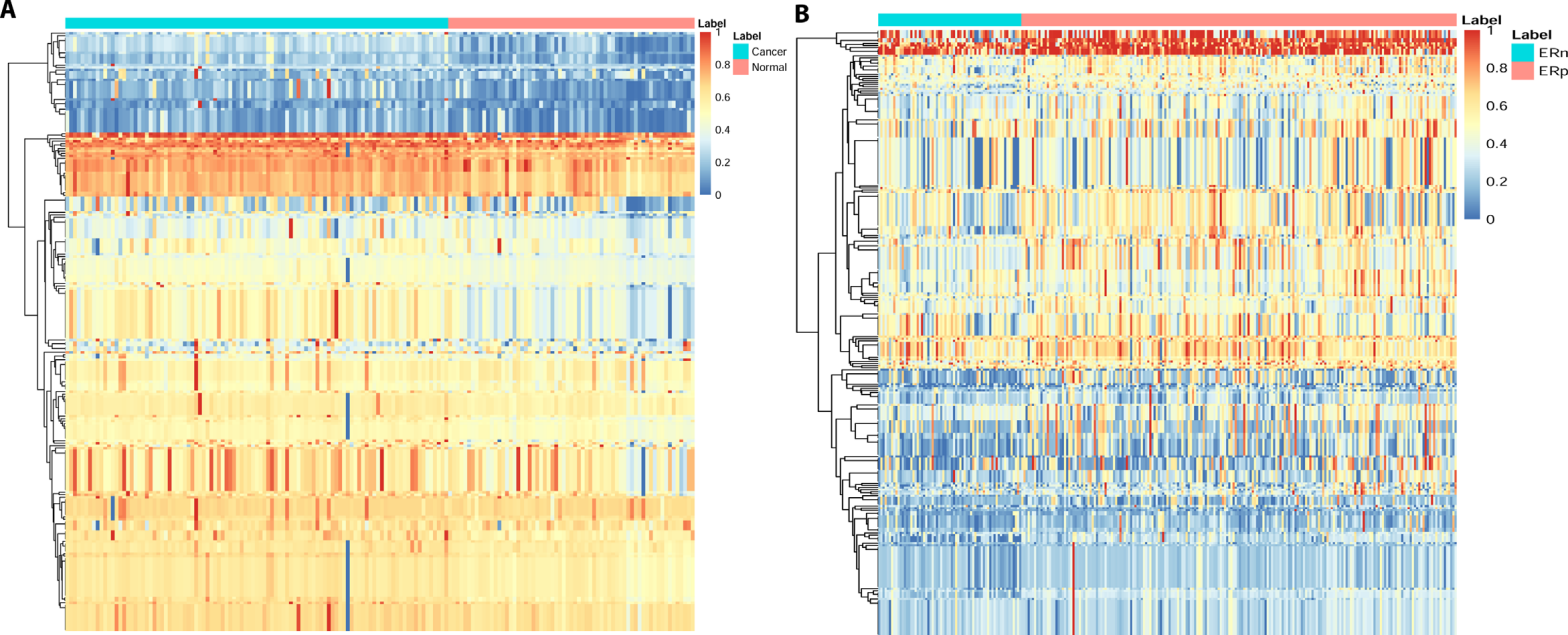
Heat map of the individual-based pathway dysregulation scores (PDS) generated by Lilikoi. The rows are the pathway IDs, and the columns are the patients separated by group. PDS score is a personalized pathway metric ranging from 0 to 1. Higher PDS score indicates more dysregulation. **(A)** Dataset 1, breast cancer vs healthy control plasma samples. **(B)** Dataset 2, ER+ vs ER− breast cancer tissue samples.

After transforming metabolites to standardized IDs, the metabolomics profile of the training set is transformed to a pathway-based profile through module 2: dimension transformation, with additional phenotype input (cancer/control) also provided by users. In the first dataset, the metabolites are mapped to 224 pathways, and the resulting PDS score (ranging from 0 to 1) based matrix with 224 pathways (rows) and 207 patients (columns) are shown in Fig. 3A. The hierarchical clustering analysis on the pathways further demonstrates that the cancer and control samples are distinguishable by several pathway clusters. For example, the first pathway cluster in Fig. 3A includes pathways that have a low PDS score in general but higher PDS in cancer patients compared to those in control samples; a close examination reveals that they are related to sugar metabolism. One of these signature pathways is called “Warburg effect,” which is a hallmark of cancer and entails altered metabolism in cancer cells with increased glucose uptake and fermentation of glucose to lactate [29].

On the contrary, other pathway-based methods, such as MetPA [11], IMPaLA [12], and MPEA [13], are not capable of generating such individual patient-level pathway matrices. Rather, they employ enrichment analysis to compare the difference of pathways at the case vs control group level (rather than the individual level). Supplementary Table S1 shows the retrieved metabolomics pathways, their statistical enrichment test significance (*P* value and adjusted *P* value), and the number of metabolites involved in each pathway that were included in the metabolites dataset. Aminoacyl-tRNA biosynthesis (*P* = 5.8 e-09), biochemical pathways part I (*P* = 4.4 e-34), and protein digestion and absorption (*P* = 3.15 e-20) are the most significant pathways for MetPA, IMPaLA, and MEPA, respectively. Notably, although these methods retrieve significant pathways, these pathways cannot be used as the input features for downstream statistical modeling and classification in the following sections. Thus, Lilikoi is a unique pathway-based metabolomics analysis package that enables rigorous biomarker predictive modeling.

We conducted similar clustering of pathways on dataset 2 (Fig. 3B) and found similarly that ER+ and ER− samples are well separated by the pathways. Moreover, since this dataset includes all cancer samples, their PDS differences are overall less than those in the plasma dataset of cancer vs healthy samples (Fig. 3A).

### Metabolomics feature selection

**Figure 4:**
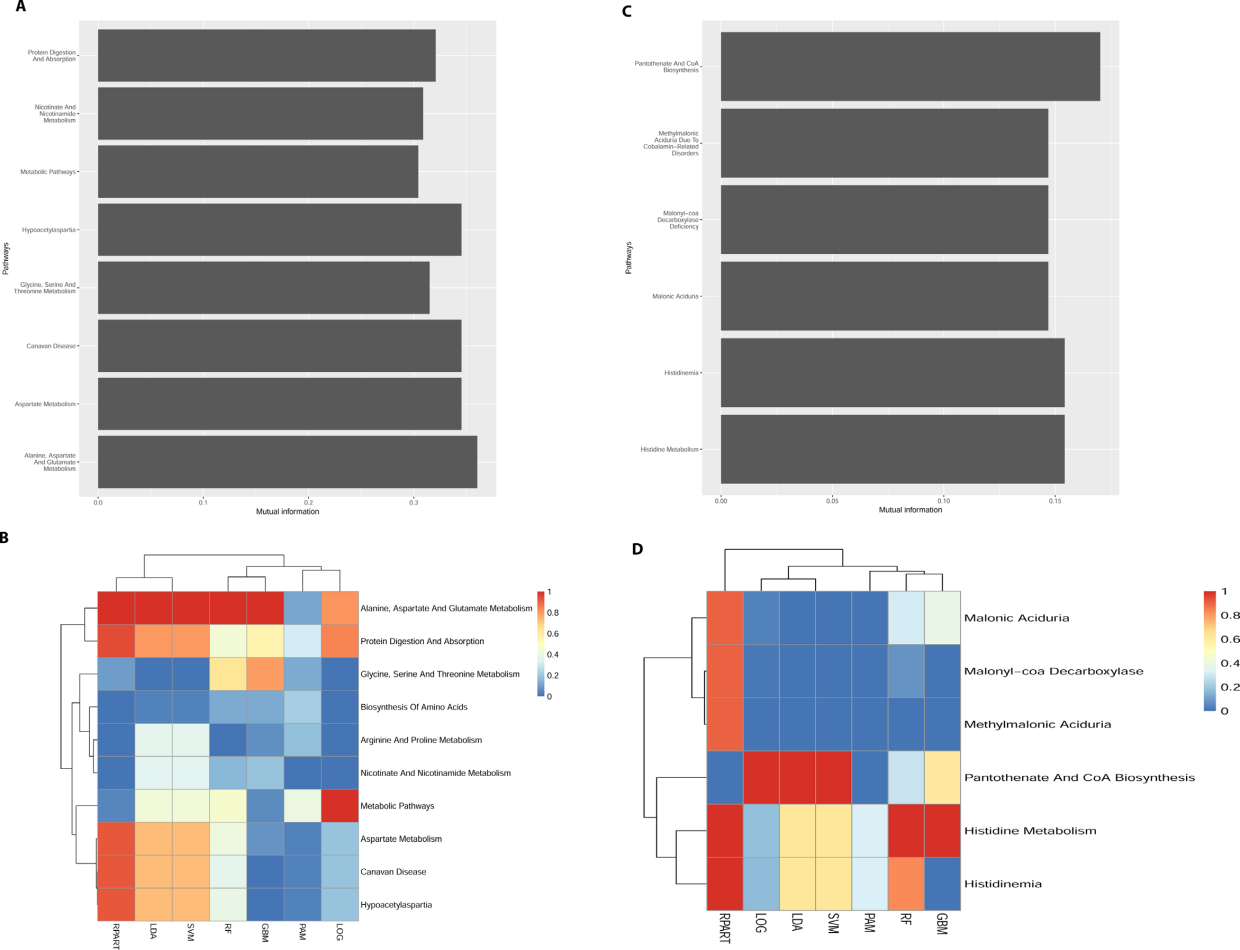
Measurements of selected pathway features in the two exemplary data sets. **(A-B)** Dataset 1, breast cancer vs healthy control plasma samples. **(C-D)** Dataset 2, ER+ vs ER− breast cancer tissue samples. **(A, C)** Selected pathway features measured by information gain, before constructing the classification models. The *x*-axis represents information gain score that measures the importance of the pathways, and the *y*-axis displays the names of pathways selected from the training data. **(B, D)** Heat map of selected pathway features measured by importance score, after constructing the classification models. The importance score is ranged from 0 (blue color) to 1 (red color). The seven machine learning methods from left to right are recursive partitioning and regression analysis (RPART), linear discriminate analysis (LDA), support vector machine (SVM), random forest (RF), generalized boosted model (GBM), prediction analysis for microarray (PAM), and logistic regression (LOG).

The next step is the feature selection module, using the PDS matrix and phenotypes of the training set as input. We then split each dataset into 80% training and 20% hold-out testing set. The user can choose either information gain (mutual information) or gain ratio to select key pathway attributes. Lilikoi plots a bar plot of selected features and their relevance to phenotype labels. Lilikoi enables the output of information gain score, a measure of feature relevance to phenotype for each selected attribute (Fig. 4A and 4C). The information gain score is a nonparametric, model-free score between 0 and 1. It can be used to rank all features relevant to the classification. The higher the value, the more relevant the feature is to classification. In dataset 1, nine pathways are identified as feature pathways in the plasma training set. Among them, alanine, aspartate, and glutamate metabolism stand out as the pathway most relevant to the disease phenotype, with the highest information gain score of 0.36. Aspartate metabolism is the second most important pathway, with the information gain score of 0.29. These rankings are in accordance with the previous report, where a lower level of plasma aspartate is one of the most important metabolomics feature in human breast cancer [30].

In dataset 2, six pathways are identified as feature pathways in the breast tissue training set (Fig. 4C). Among them, pantothenate and CoA biosynthesis stand out as the pathways most relevant to the disease phenotype, with the highest information gain score of 0.19. Histidine metabolism is the second most important pathway, with the information gain score of 0.16. Interestingly, none of these pathways were mentioned in the original study that was focused on metabolite-level analysis [27]. To investigate if our pathway analysis truly reveals interesting metabolic changes, we looked into the metabolites that are associated with these top pathways in the dataset 2. Impressively, beta-alanine is the metabolite associated with all six feature pathways, and it was reported in the original study as the signature metabolite to differentiate ER+ and ER− samples. While many other pathways such as GABA-transaminase deficiency and ureidopropionase deficiency also have beta-alanine as part of metabolite, the fact that only the six pathways are selected as features indicates that they are more relative to ER+ and ER− separation.

### Model construction and validation

**Figure 5:**
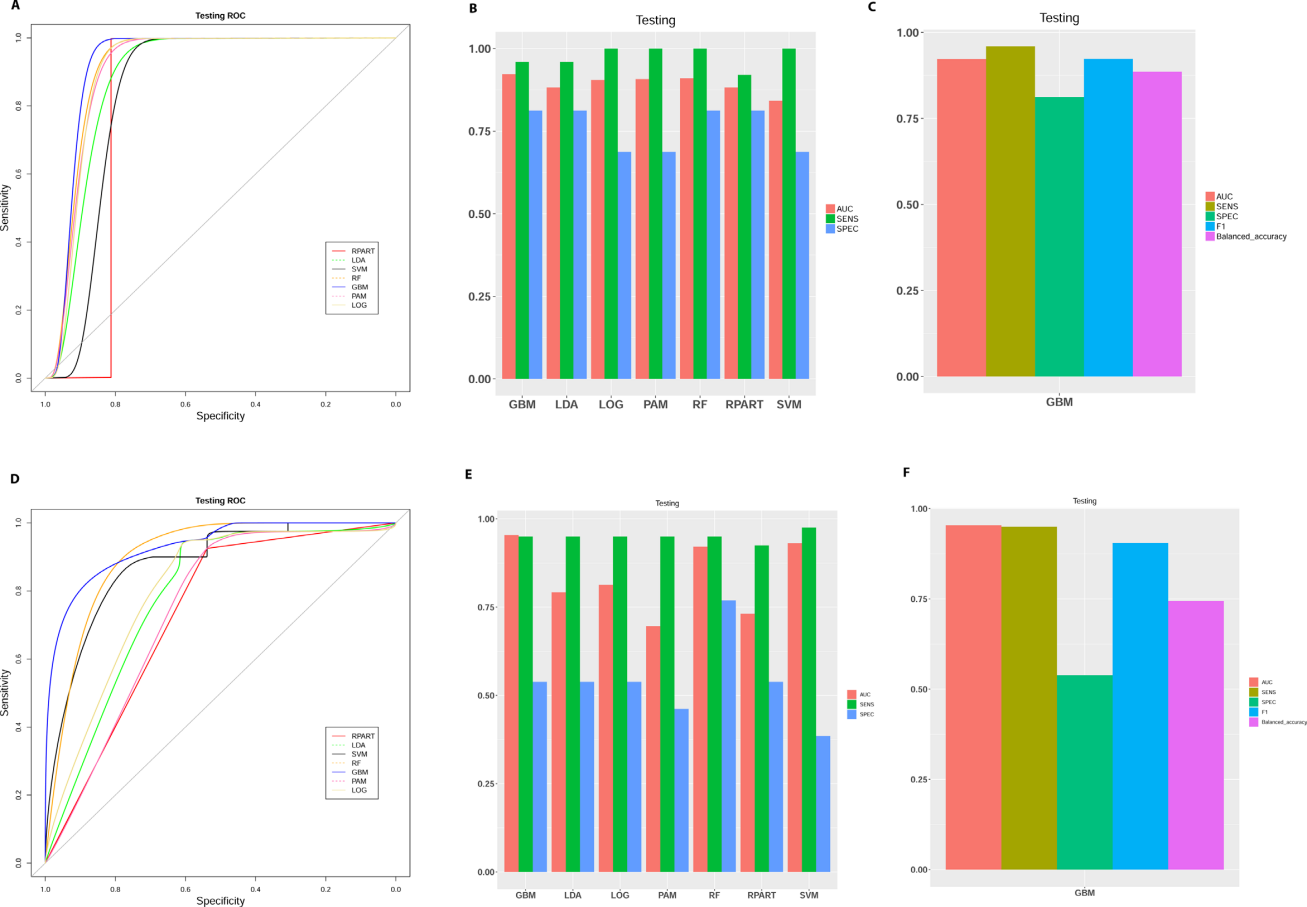
Model evaluation on the two exemplary datasets. **(A-C)** Dataset 1, breast cancer vs healthy control plasma samples. **(D-F)** Dataset 2, ER+ vs ER− breast cancer tissue samples. **(A, D)** ROC curves of the breast cancer diagnosis testing set, obtained from seven classification algorithms: recursive partitioning and regression analysis (RPART), linear discriminate analysis (LDA), support vector machine (SVM), random forest (RF), generalized boosted model (GBM), prediction analysis for microarray (PAM), and logistic regression (LOG). **(B, E)** Metrics (AUC, sensitivity, specificity, and F-1 statistic) to measure the performance of classification on training or testing data. **(C, F)** Metrics of the best-performing model on testing data, based on the criteria chosen by the user (AUC in this case).

The last step is classification model construction and prediction. This module builds a model from the selected pathway features and allows the users to select among seven different classification algorithms with n-fold cross-validation. The user can compare performance measurements and choose the best classifier as the model of choice (Fig. 5). This module generates two types of figures: a plot of ROC curves (Fig. 5A, D) that present the overall model performance on the testing dataset and a second bar plot (Fig. 5B, E) that illustrates the values of additional performance metrics (e.g., AUC, SEN, SPEC) of the testing data. In addition, Fig. 5C shows the performance metrics generated from the best-performing model, using a user-selected metric. In this example, we use AUC as the metric to select the best model, and GBM algorithm yields the best performance (Fig. 5C, F).

Next, we checked the importance of these pathway features (Fig. 4A, C) relative to each classification model (Fig. 4B, D). For dataset 1, interestingly, all machine learning methods place consistently high importance scores to pathway alanine, aspartate, and glutamate metabolism and protein digestion and absorption, supporting its significance (Fig. 4B). The importance of alanine, aspartate, and glutamate metabolism has been confirmed before [30]. On the other hand, some pathways had discrepant importance scores ranked by the machine learning methods, such as aspartate metabolism and hypoacetylaspartia (a defect in L-aspartate-N-acetyltransferase resulting in a strongly decreased concentration of N-acetyl-L-aspartic acid). For dataset 2, less consistency is found among different machine learning methods on pantothenate and the CoA biosynthesis pathway, although it is the highest ranking pathway by three machine learning methods (Fig. 4D).

### Model calibration by addressing confounding

**Figure 6:**
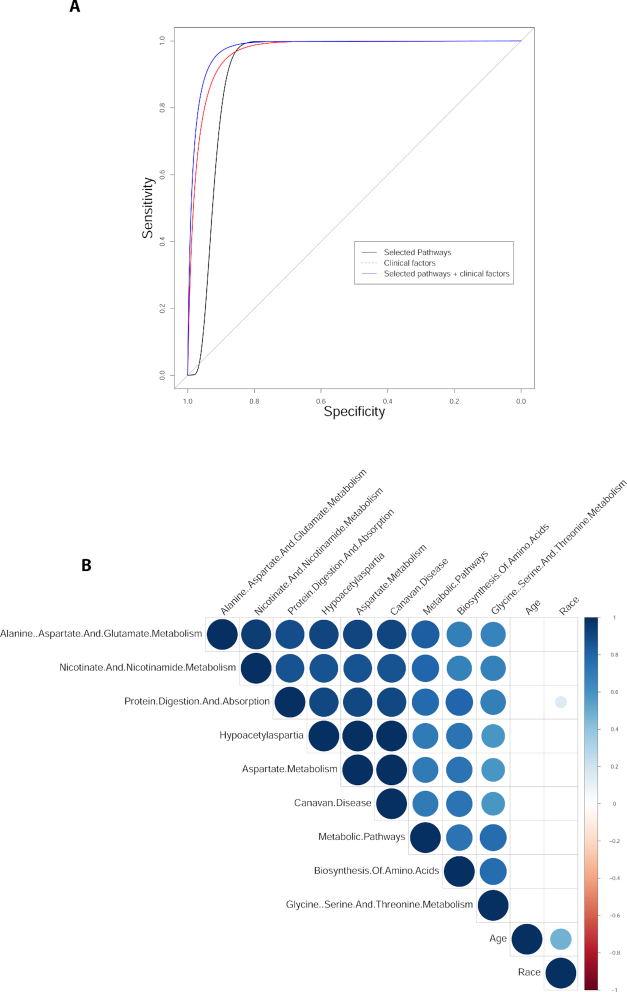
Calibration of metabolomics model on dataset 1 by confounding. **(A)** ROC curves of metabolomics only, clinical data only, and the metabolomics clinical combined model. **(B)** Correlation coefficients among demographic/physiologic factors and the metabolomics data. Blue indicates positive correlations and red indicates negative correlations.

Adjusting the fitted model using the clinical factors (if available) is a critical step in metabolomics-based biomarker research. In this step, Lilikoi builds three models using metabolomics data, or clinical data, or the combination of the two types of data, and plots their ROC curves on the corresponding testing sets (Fig. 6A). Model 1 (black solid curve) is created using the selected pathways from the features selection module; model 2 (red dashed curve) uses the clinical factors selected by the user; and model 3 (blue solid curve) is created by combining both selected pathways and selected clinical factors. In this example, the clinical factors impose significant confounding in classification, and age is the primary contributor in the clinical model (data not shown). To understand the relationships among the selected pathways and the clinical factors, a correlation heat map is plotted in Fig. 6B.

## Discussion

Metabolomics biomarker discoveries have gained an increasing amount of attention recently in a variety of applications such as disease diagnosis and progression. Currently, most of the biomarker features in the metabolomics field are represented as individual metabolites, which suffer from inconsistency among studies. On the other hand, most pathway-based methods in the metabolomics field are not personalized and they are merely used for graphical mapping and enrichment analysis. None of these metabolomics pathway-based tools employ pathways as features for downstream biomarker modeling. Lilikoi addresses all of these issues with personalized pathway deregulation measurements (PDS scores) and offers a standardized classification model for biomarker prediction. Compared to the traditional way of identifying individual metabolites as biomarkers, pathway-based biomarkers are more tolerant of population heterogeneity. Additional advantages of Lilikoi include the flexibility of its feature selection methods, the use of various machine learning classification algorithms, and its automatic tuning of parameters to generate the best model for a specific algorithm.

As an R package that will undergo active improvements, Lilikoi can potentially benefit from other technical tweaking. Currently, a small percentage (20%) of the metabolites still cannot be mapped to the standard names in databases. One possible reason for this mismatching is that we used the Levenshtein distance as a measure of the similarity between the user's query metabolites and the metabolites stored in Lilikoi's database. However, regardless of mapping facilitation, the first line of reporting practice is to always use metabolite standard identifiers. Lilikoi uses standard IDs such as PubChem CIDs, HMDB IDs, InChiKey or METLIN IDs for the mapping process. Additionally, although the parameters in each classification model are automatically optimized, there is no automatic algorithm (AutoML) implemented that selects the best overall classification model; rather it depends on the user's subjective preference of a machine learning method. It would be beneficial to automatically provide users with references for classification algorithm selection, without human supervision [31, 32]. We plan to use AutoML in our classification module in the future.

## Availability of source code and requirements

Project name: Lilikoi

Project home page: https://github.com/lanagarmire/lilikoi

Operating system(s): Windows and Linux

Programming language: e.g., R

Other requirements: e.g., R3.5.1

License: GPLv3

The R package for Lilikoi is accessible at https://cran.r-project.org/web/packages/lilikoi/. The source code is also freely available under the GPLv3 license through the github repository at: https://github.com/lanagarmire/lilikoi. We made a web tool for Lilikoi to facilitate programming-free use of the package. The GUI link is: Lilikoi.garmiregroup.org. Additionally, Docker image and binder image can be accessed at: https://mybinder.org/v2/gh/FADHLyemen/lilikoi_Fadhl/master

## Availability of supporting data

Additional supporting data, which includes R package scripts and snapshots of the code, including Docker image and binder image, are available in the *GigaScience* repository, GigaDB [33].

## Additional files

Table S1: the enrichment analysis results of other pathway methods on data set 1: MetPA, IMPaLA, and MPEA.

## Abbreviations

AUC: area under the curve; CRAN: The Comperhensive R Archive Network; ER: estrogen receptor; GBM: generalized boosted model; GSEA: gene set enrichment analysis; HDMB:; KEGG: Kyoto Encyclopedia of Genes and Genomes; IMPaLA: pathway analysis with transcriptomics and metabolomics data; LDA: linear discriminate analysis; LOG: logistic regression; METLIN: metabolite and tandem MS database; MPEA: metabolite pathway enrichment analysis; MetPA: a web-based metabolomics tool for pathway analysis and visualization; PAM: prediction analysis for microarray; PDS: pathway deregulation score; RF: random forest; ROC: receiver operating characteristic; RPART: recursive partitioning and regression analysis; SEN: sensitivity; SPEC: specificity; SVM: support vector machine.

## Competing interests

The authors declare that they have no competing interests.

## Funding

This research was supported by grants K01ES025434 awarded by National Institute of Enviromental Health Sciences (NIEHS) through funds provided by the trans-NIH Big Data to Knowledge (BD2K) initiative (www.bd2k.nih.gov), P20 COBRE GM103457 awarded by National Institute of Health/ National Institue of General Medical Sciences (NIH/NIGMS), R01 LM012373 awarded by National Library of Medicine (NLM), R01 HD084633 awarded by Eunice Kennedy Shriver National Institue of Health (NICHD) to L.X.G.

## Author Contributions

LXG envisioned the project, obtained funding, designed and supervised the project and data analysis. SJH and FMA implemented the package. FMA packaged the software, wrote documentations & instructions and generated Figures 2-6. SJH wrote the majority of the draft with help from FMA, and generated Figure 1. BY tested the package and wrote the script to update the metabolomics database. HA developed the Shiny version of the package. All authors have read, revised, and approved the manuscript.

## Supplementary Material

Response_to_Reviewer_Comments_Original_Submission.pdf

Response_to_Reviewer_Comments_Revision_1.pdf

Reviewer_1_Report_(Original_Submission) -- Jeff Xia 4/11/2018 Reviewed

Reviewer_1_Report_Revision_1 -- Jeff Xia 8/3/2018 Reviewed

Reviewer_3_Report_(Original_Submission) -- Zhibo Yang 4/25/2018 Reviewed

Reviewer_3_Report_Revision_1 -- Zhibo Yang 7/30/2018 Reviewed

giy136_Supplement_Table_S1
