# Supplementary material for: Lilikoi: an R package for personalized pathway-based classification modeling using metabolomics data
Source: Gigascience. 2018 Dec 10;7(12):giy136. doi: 10.1093/gigascience/giy136 (PMC6290884; doi:10.1093/gigascience/giy136)
Supplement: Response_to_Reviewer_Comments_Original_Submission.pdf [file giy136_response_to_reviewer_comments_original_submission.pdf]

Author's Response To Reviewer Comments

Close

Dear Editor Prof. Nicole Nogoy,

Thank you very much for considering our revised manuscript entitled: “Lilikoi: an R package for personalized pathway-based classification modeling using metabolomics data”.

We sincerely thank the reviewers for their very constructive suggestions which have significantly reshaped this revision. We have addressed in full every single question of the reviewers, point by point in the attached file, along with the revised manuscript.
We registered our package in SciCrunch database (RRID: SCR_016361) for the sake of reproducibility and re-use of Lilikoi.
We hope the manuscript is satisfying. Please feel free to address all correspondence concerning this manuscript to me at lgarmire@cc.hawaii.edu.

Sincerely,

Lana Garmire, PhD
Associate Professor (with tenure)
Epidemiology Program, University of Hawaii Cancer Center

Answers to reviewers

Reviewer’s comments are in black. Our responses are in blue.

Reviewer #1:

Al-Akwaa et al described an R package for personalized pathway-based classification modeling using metabolomics data. The package contains four main components for compound name mapping, converting from metabolite profiles to pathway profiles, identification of significant pathway features, and classification/prediction. This is a comprehensive pipeline built upon many other R packages. However, after careful evaluation, the reviewer feels that the scientific contribution of this R package is limited and quality of the work is insufficient.

1) Lack of novel contribution.
It is clear from the manuscript that a key contribution of the package is to use the 'pathifier' package to convert from metabolite concentration profiles to pathway profiles based on PDS scores. The authors have shown how such approach was able to obtain novel insights compared to other approaches. However, a) The algorithm is not a novel contribution. b) In terms of novel application (or novel biological insights), the authors' group already demonstrated this in their 2016 Genome Medicine paper "Novel personalized pathway-based metabolomics models reveal key metabolic pathways for breast cancer diagnosis".

Thank you for your comments.

To address your question on novel algorithm contribution, please allow us to point out that

Lilikoi fills a void that currently there is no complete software package that uses pathway as metabolomic features for classification modeling. Besides using pathifier method as done in 2016 Genome Medicine paper, Lilikoi has many new functions that the original paper did not implement or automate, such as metabolites mapping, feature selection, classification, and handling confounding factors besides metabolomics data. This is the first complete package in this regard, rather than an R script that can partially do pathway based classification semi-automatically as done in 2016 Genome Medicine paper. This package is to address the feedbacks from readers of the original research paper.

To address your question on novel application, we now have included a second metabolomics dataset second data set. It consists of 271 breast cancer tissue samples (204 ER+ and 67 ER−) collected from a biobank [1]. We amended the results section, as well as adding new Fig 3B, Fig 4 C, D and Fig 5 D, E, F.

Reference:
[1]:Budczies, J.; Denkert, C.; Muller, B. M.; Brockmoller, S. F.; Klauschen, F.; Gyorffy, B.; Dietel, M.;Richter-Ehrenstein, C.; Marten, U.; Salek, R. M.; Griffin, J. L.; Hilvo, M.; Oresic, M.; Wohlgemuth, G.;Fiehn, O. Remodeling of central metabolism in invasive breast cancer compared to normal breast tissue - a GC-TOFMS based metabolomics study BMC Genomics 2012, 13, 334 DOI: 10.1186/1471-2164-13-334

2) Lack of user friendliness.
a) Users need to know R in order to use this package. However, for researchers familiar with R, they can almost do all these tasks without using this pipeline, as almost all its component packages are already available. Such a pipeline should ideally be implemented to have graphical user interface (GUI) so other people (who don't know R) can still use.

Thank you for the suggestion. We have developed a Shiny GUI version of Lilikoi, so users with no programing background can benefit from it. The GUI link:
Lilikoi.garmiregroup.org

b) Even as an R package, the standard publication route is CRAN, with built-in unit testing and vignette tutorial. Installing R package from CRAN is very straightforward as compared to GitHub. In addition, publishing on CRAN also facilitates quality assurance and long-term
maintenance.

Thank you and points well taken. We have submitted Lilikoi to the CRAN repository. Once approved, the R package with reference manual and vignettes will be accessible from the link: https://cran.r-project.org/web/packages/lilikoi/

Reviewer #2:
Hi, I asked the editor to forward my comments to the authors, but I'd like to opt out of the open peer review policy of the journal.

Comments - The authors describe an R package, that can map preprocessed metabolomics data to networks, and allows to compare/statistically analyse difference between these sample-based networks. The package installed as described without problems. The package content is not necessarily very novel, but likely there is a niche and application for this.

I don't like the concept of the FeatureMapper as part of the data analysis pipeline. This approach has the danger that the data generating groups do not improve their reporting with proper compound accession numbers, and leave the mapping issue to the data analysis people. From a chemists' perspective, the Levenshtein distance in the third stage really is only a last resort, and no proper mapping. Instead, the feature mapper should (also) be described as a way to improve the way biologists acquire and store the data. All metabolomics data. The manuscript should mention that virtually no downstream metabolomics data analysis is possible without metabolite identifiers (regardless whether that's PubChem CIDs, HMDB IDs, InChiKey, ...) and chemical names should be treated only as secondary human friendly identifiers.

Thank you for your feedback.

We absolutely agree that reporting with proper compound accession numbers is essential for metabolomics studies. This was exactly the reason that prompted us to have FeatureMapper in the pipeline, since in the original Genome Medicine paper we had to ask an undergraduate student to do labor intensive manual matching for some metabolites.

We took the reviewer's suggestion and stressed the importance in this aspect, that virtually no downstream metabolomics data analysis is possible without metabolite identifiers. We caution users that fuzzy matching should be used as the last resort, and standard naming should be the best practice. Please see our highlighted sections in Methods, Results and Discussion sections of the revision.

Even when standard IDs are provided, bioinformaticians spend a lot of times to convert IDs in practice. If the user provides one set of ID (eg. HMDB ID), the featureMapper can convert it to another set of ID (eg. PubChem ID). This provides them convenience, thus we keep this section.

When describing the FeatureMapper, make sure to cite the Chemical Translation Service (CTS) and the BridgeDB R Package.

Thank you. We updated the Methods section (Feature Mapper subsection) to include these citations as suggested:

"A few tools have been developed to address the issue of identifiers mapping. The Chemical Translation Service (CTS) is a web based tool for metabolites ID conversion [15]. BridgeDb is another R package which support genes, protein and metabolites identifier mapping [16]."

Please mention a bit more about the characteristics of the example data set, e.g. that it is available as PR000284.

Sure. We improved the manuscript to include more information:

"For demonstration, we present a metabolomics data set from the City of Hope Hospital (COH) that was published earlier [4]. This dataset is composed of 207 samples from plasma (126 cases and 81 controls). The details of the data are summarized in our previous work [4]. This dataset can be downloaded from Metabolomics workbench

(http://www.metabolomicsworkbench.org) with project ID PR000284."

The availability of Lilikoi on github is a first step, but R packages are poorly findable on github.
The package should be submitted to CRAN or Bioconductor together with this publication.

Great suggestion! We have submitted Lilikoi to the CRAN repository. Once approved, the R package with reference manual and vignettes will be accessible from the link: https://cran.r-project.org/web/packages/lilikoi/

Lilikoi embeds 18000 metabolites from several sources. What is the update strategy ? Will it be updated on a defined e.g. quarterly schedule? Is there a description how the user can update the database?

Lilikoi provides a default database, it also allows updated database if the user prefers. We added a lilikoi.updateDB method, with which users can call to pull the latest curated databases from Lilikoi's github repository. We plan to update the database periodically (eg. semi-annually).

I saw that the github also contains a jupyter notebook. Can you check if the notebook works with https://mybinder.org/ ? Could you provide a Docker container based
on e.g. https://hub.docker.com/r/jupyter/notebook/ to execute the demo notebooks ?

Thank you. Yes, we checked the notebook on mybinder.org and here is a live notebook:
https://mybinder.org/v2/gh/FADHLyemen/lilikoi_Fadhl/master

Also, we pushed the Lilikoi's docker image in the docker hub repository. Users can pull Liliko using the command below:
docker pull fadhlyemen/lilikoi
And run it using the command below:
docker run -d --rm -ti -p 5001:8888 fadhlyemen/lilikoi start-notebook.sh --NotebookApp.token="

Section on "model calibration", line 25: Are the samples from age matched patients? I am curious here. From the brief reading it looks as if samples from older patients are more likely to be affected. Regardless of the network created. That would rather be an issue with the study, and not the R package.

Yes, in the original study design had some issue, where age is a confounder (not matched between case and control). This is exactly the motivation behind Lilikoi's model adjustment: by including confounding factors in the model. In some cases, it may be difficult to know what the confounders are beforehand, and such model calibration can come to rescue.

Typos:
- line 13: matabolism standS out as
- line 15: (remove: And) Aspartate metabolism is ...

Thank you. We have corrected these typos.

Reviewer #3:

The authors have developed a comprehensive R package for pathway analysis using metabolomics data. This package contains four modules that are integrated to improve the capability and flexibility for metabolomics data analysis and biomarker prediction. Compared with many existing metabolomics analysis tools, this R package is more comprehensive. The authors demonstrated the capability of this package usinwell prepared. However, some descriptions are unclear. This manuscript needs a major revision.

Major concerns:

(1) In "Method", the second module (Dimension transformation) transforms metabolite profiles to a pathway deregulation score (PDS) matrix, which plays important role to obtain pathway-level metabolomics. PDS seems to be new in the related studies. Although there is a very brief description, it is unclear how PDS is calculated.

Thank you for the comment. We have updated the manuscript to give more description of how PDS was calculated, as follows:

“The PDS score DP(i) was calculated for each pathway P and each sample i, based on the intensities of the metabolites in pathway P. This score estimates the extent to which the pathway P in sample i deviates from the control. Briefly, in the high dimensional space dP made of metabolite vectors (where each metabolite belongs to pathway P), all samples form data cloud where sample i is a data point xi. The principle curve SP′ in this space dP is then calculated using Hastie and Stuetzle’s algorithm. For each sample, the data point xi is projected onto the principle curve SP′. The deregulation score DP(i) of sample i is then defined as the distance from the start of the principle curve to the projected point on this curve.

(2) In "Feature selection", two major feature selection algorithms (information gain and gain ratio) are provided; and the later one is more suitable to avoid over-fitting issues. Does this R package provide a measure of over-fitting, so users can select the appropriate methods?

Thanks for the great suggestion! Yes it is possible to tell whether the selected features caused model overfitting, by looking at the difference of accuracies between the training and testing sets. If the accuracy of the training set is much larger than that of the test set, this indicates over-fitting. We have added this suggestion in the Methods section (under Feature Selection subsection).
In the GUI version of Lilikoi, we updated training and testing barplot with accuracy values, which guides the users to decide which feature selection and classification method to choose in order to minimize over-fitting.
(3) In "Standardization and mapping of metabolomics IDs", the authors mentioned that "Lilikoi embeds comprehensive databases including over 18,000 metabolites and 100,000 synonyms, in accordance with other types of input is". It is unclear if the embedded databases need to be updated by users.

We clarified this in the revision. Lilikoi provides a default database, also allows updated database if the user prefers. We added a lilikoi.updateDB method, with which users can call

to pull the latest curated databases from Lilikoi’s github repository.

(4) A large number of acronyms are used in this article; however, many of them are not spelled out before being used. For example, those seven machine learning methods, SEN, SPEC…

Our apologies. We have updated the manuscript with the full names for these acronyms.

(5) In "Metabolomics feature selection", the "information gain score" has been used for the analysis. This term was not defined in the text. It is also unclear the meaning of the numbers (e.g., 0.36 and 0.29 used in the discussion).

Thanks for pointing them out. We have defined the information gain score in the revised manuscript as follows:

“The information gain score is a nonparametric, model-free score between 0 and 1. It can be used to rank all features relevant to the classification. The higher the value, the more relevant the feature is to classification.

0.36 and 0.29 there indicates how relevant that particular pathway feature is to breast cancer diagnosis, based on the dataset.

(6) Resolution of figures 3-5 needs to be improved.

Thank you. We have updated these figures with higher resolution.

Minor corrections

Most part of this manuscript are well written; however, some sentences need to be rephrased.

(1) In Introduction, the third paragraph. "The common feature of these methods is that they use metabolites as biological entities to summarize to pathway-level statistics at group level (separated by states), and then perform enrichment analysis (such as over-representation analysis and Gene-Set-Enrichment-Analysis) in order calculate the overrepresentation of pathways in one group versus the other group."; this sentence has some grammar errors and doesn't read smoothly.

Thank you. We have rephrased the above sentence as the following:

The common feature of these methods is that they summarize metabolite-level intensities to pathway-level statistics at group level (separated by states), and then perform enrichment analysis (such as over-representation analysis and Gene-Set-Enrichment-Analysis). As a result, overrepresentation of pathways in one group versus the other group is estimated.

(2) In "Model calibration by addressing confounding". "In this step, Lilikoi build three models on the training data set and plots the ROC curves on the testing set (Figure 6-A)." needs to be rephrased.

Thank you. We have rephrased the above sentence to be:

“In this step, Lilikoi builds three models using metabolomics data, or clinical data, or the combination of the two types of data, and plots their ROC curves on the corresponding testing sets (Figure 6-A).”

Close
