# Supplementary material for: Lilikoi: an R package for personalized pathway-based classification modeling using metabolomics data
Source: Gigascience. 2018 Dec 10;7(12):giy136. doi: 10.1093/gigascience/giy136 (PMC6290884; doi:10.1093/gigascience/giy136)
Supplement: Response_to_Reviewer_Comments_Revision_1.pdf [file giy136_response_to_reviewer_comments_revision_1.pdf]

Author's Response To Reviewer Comments

Close

Dear Editor Dr. Nicole Nogoy,

Thank you very much for giving us a minor revision decision on the manuscript entitled: "Lilikoi: an R package for personalized pathway-based classification modeling using metabolomics data".

We sincerely thank the reviewers for their very constructive suggestions. We have addressed in full every single question of the reviewers, point by point in the attached file, along with the revised manuscript.
We hope the manuscript is now acceptable for publishing. Please feel free to address all correspondence concerning this manuscript to lgarmire@med.umich.edu

Sincerely,

Lana Garmire
Associate Professor
Department of Computational Medicine and Bioinformatics
Medical School
University of Michigan

Answers to reviewers

Reviewer's comments are in black. Our responses are in blue.

Reviewer #1:

The authors have carefully addressed my comments. I recommend for publication.
I have one minor comment: the Shiny app appears to be functional and well designed. However, better documentations are needed to make it more accessible.

Thank you for your comments.
We added a user guide which includes all of the Lilikoi steps and modules.
https://github.com/lanagarmire/lilikoi/blob/master/lilikoi_shiny_user_manual.pdf

Reviewer #2:
The authors addressed all of my comments of the first review.
Thank you.

Some of the new text snippets in yellow are poorly written, and need some careful language checking.

We apologize, we corrected them.

The explanation l13: "Reporting proper compounds with standard accession numbers is paramount important, and virtually downstream metabolomics data analysis is impossible without proper metabolite identifiers"
is lacking WHY that is so important. People like the poor undergrad student know for sure, but please mention that only with a unique identifier it is possible to directly link to a metabolite, rather than searching for a name.

Thank you. We updated this section to include why it is important to have unique accession numbers for the metabolites.

Actually, the same is true for pathways. In your "Table S1.xlsx" you have Pathway NAMES. Without identifier have no idea how you counted the #20 metabolites there, or where to get a picture/map of the pathway. In fact, https://www.genome.jp/dbget-bin/www_bget?hsa00970 has 52 Compounds associated. (Or is it that only #20 out of #52 have been part of the input data?) That's why we need Identifiers for ALL things. Please add pathway identifiers to the supplemental table(s).

Thank you. We updated Table S1 with all pathways identifiers.
Yes, pathway “Aminoacyl-tRNA biosynthesis” includes 20 metabolites as part of our input data. We updated the text to make it clear.
“Table S1 shows the retrieved metabolomics pathways, their statistical enrichment test significance (p-value and adjusted p-value), and the number of metabolites involved in each pathway that were included in the metabolites dataset.”

The lilikoi package has indeed been accepted in CRAN.
The mybinder example is great for trying the features without installing the package.

Thank you.

The code of the Shiny App should go into the package itself, allowing to run either the scripted version or the GUI. That also keeps GUI and Code better in Sync.

Good point. We added the Shiny app into the Github repository and will go into our CRAN app in the next release. Users can run the Shiny app locally using the commands below:

```
git clone https://github.com/lanagarmire/lilikoi --branch shiny
R -e "shiny::runApp('.')"
```

The comment l7: "beta-alanine is the metabolite associated with all six pathways" is interesting. Are there other pathways with beta-alanine that are not enriched? That could indicate that this analysis helps to focus on those beta-alanin-containing pathways that are relevant. If beta-alanine is associated to just these six pathways, that is not much information added. Please expand that interpretation in the manuscript.

Thank you. Yes, other pathways with beta-alanine but lower feature scores are not enriched. We expand the interpretation in the manuscript.
For these six pathways, we set the feature score threshold to 0.35. When we lowered the threshold (0.30), new pathways including Carnosinuria Carnosinemia, GABA-Transaminase

Deficiency and Ureidopropionase Deficiency are selected, and all of these three new pathways include beta-alanine.
Typos:

l42: Leftover citation: [26]For demonstration
l20: Additionally, Decker image
Thanks. We corrected these typos.

Close
