# Supplementary material for: Lilikoi: an R package for personalized pathway-based classification modeling using metabolomics data
Source: Gigascience. 2018 Dec 10;7(12):giy136. doi: 10.1093/gigascience/giy136 (PMC6290884; doi:10.1093/gigascience/giy136)
Supplement: Reviewer_3_Report_(Original_Submission) -- Zhibo Yang — 4/25/2018 Reviewed [file giy136_reviewer_3_report_(original_submission).pdf]

Reviewer Report

Title: **Lilikoi: an R package for personalized pathway-based classification modeling using metabolomics data**

Version: **Original Submission** **Date:** 4/25/2018

Reviewer name **Zhibo Yang**

Reviewer Comments to Author:

The authors have developed a comprehensive R package for pathway analysis using metabolomics data. This package contains four modules that are integrated to improve the capability and flexibility for metabolomics data analysis and biomarker prediction. Compared with many existing metabolomics analysis tools, this R package is more comprehensive. The authors demonstrated the capability of this package using previously published data. In general, this manuscript has been well prepared. However, some descriptions are unclear. This manuscript needs a major revision.

Major concerns:

(1) In "Method", the second module (Dimension transformation) transforms metabolite profiles to a pathway deregulation score (PDS) matrix, which plays important role to obtain pathway-level metabolomics. PDS seems to be new in the related studies. Although there is a very brief description, it is unclear how PDS is calculated.

(2) In "Feature selection", two major feature selection algorithms (information gain and gain ratio) are provided; and the later one is more suitable to avoid over-fitting issues. Does this R package provide a measure of over-fitting, so users can select the appropriate methods?

(3) In "Standardization and mapping of metabolomics IDs", the authors mentioned that "Lilikoi embeds comprehensive databases including over 18,000 metabolites and 100,000 synonyms, in accordance with other types of input IDs". It is unclear if the embedded databases need to be updated by users.

(4) A large number of acronyms are used in this article; however, many of them are not spelled out before being used. For example, those seven machine learning methods, SEN, SPEC…

(5) In "Metabolomics feature selection", the "information gain score" has been used for the analysis. This term was not defined in the text. It is also unclear the meaning of the numbers (e.g., 0.36 and 0.29 used in the discussion).

(6) Resolution of figures 3-5 needs to be improved.

Minor corrections

Most part of this manuscript are well written; however, some sentences need to be rephrased. .

(1) In Introduction, the third paragraph. "The common feature of these methods is that they use metabolites as biological entities to summarize to pathway-level statistics at group level (separated by states), and then perform enrichment analysis (such as over-representation analysis and Gene-Set-Enrichment-Analysis) in order calculate the overrepresentation of pathways in one group versus the other group."; this sentence has some grammar errors and doesn't read smoothly.

(2) In "Model calibration by addressing confounding". "In this step, Lilikoi build three models on the training data set and plots the ROC curves on the testing set (Figure 6-A)." needs to be rephrased.

Level of Interest

Please indicate how interesting you found the manuscript: Choose an item.

Quality of Written English

Please indicate the quality of language in the manuscript: Choose an item.

Declaration of Competing Interests

Please complete a declaration of competing interests, considering the following questions:

- Have you in the past five years received reimbursements, fees, funding, or salary from an organisation that may in any way gain or lose financially from the publication of this manuscript, either now or in the future?
- Do you hold any stocks or shares in an organisation that may in any way gain or lose financially from the publication of this manuscript, either now or in the future?
- Do you hold or are you currently applying for any patents relating to the content of the manuscript?
- Have you received reimbursements, fees, funding, or salary from an organization that holds or has applied for patents relating to the content of the manuscript?
- Do you have any other financial competing interests?
- Do you have any non-financial competing interests in relation to this paper?

If you can answer no to all of the above, write 'I declare that I have no competing interests' below. If your reply is yes to any, please give details below.

I declare that I have no competing interests

I agree to the open peer review policy of the journal. I understand that my name will be included on my report to the authors and, if the manuscript is accepted for publication, my named report including any attachments I upload will be posted on the website along with the authors' responses. I agree for my report to be made available under an Open Access Creative Commons CC-BY license (http://creativecommons.org/licenses/by/4.0/). I understand that any comments which I do not wish to be included in my named report can be included as confidential comments to the editors, which will not be published.

I agree to the open peer review policy of the journal

To further support our reviewers, we have joined with Publons, where you can gain additional credit to further highlight your hard work (see: https://publons.com/journal/530/gigascience). On publication of this paper, your review will be automatically added to Publons, you can then choose whether or not to claim your Publons credit. I understand this statement. Yes
